# Clinical Holistic Medicine: Induction of Spontaneous Remission of Cancer by Recovery of the Human Character and the Purpose of Life (the Life Mission)

**DOI:** 10.1100/tsw.2004.94

**Published:** 2004-05-26

**Authors:** Søren Ventegodt, Mohammed Morad, Eytan Hyam, Joav Merrick

**Affiliations:** ^1^ The Quality of Life Research Center, Teglgårdstræde 4-8, DK-1452 Copenhagen K, Denmark and The Scandinavian Foundation for Holistic Medicine, Sandvika, Norway; ^2^ Division of Community Health, Ben Gurion University, Beer-Sheva, Israel; ^3^ Soroka University Medical Center, Clalit Health Services, Faculty of Health Sciences, Ben Gurion University, Beer-Sheva, Israel; ^4^ National Institute of Child Health and Human Development, Office of the Medical Director, Division for Mental Retardation, Ministry of Social Affairs, Jerusalem and Zusman Child Development Center, Division of Pediatrics and Community Health, Ben Gurion University, Beer-Sheva, Israel

**Keywords:** quality of life, QOL, philosophy, human development, holistic medicine, public health, life mission theory, cancer, Denmark

## Abstract

The recovery of the human character and purpose of life with consciousness-based medicine seems to be able to induce spontaneous remissions in several diseases. On two different occasions, we observed breast tumors reduced to less than half their original diameters (clinically judged) during a holistic session, when working with the patients in accordance with the holistic process theory of healing, the life mission theory, and the theory of human character. One tumor was histologically diagnosed as malign breast cancer prior to the session, while the other was under examination. As both patients had the affected regions of the breast surgically removed immediately after the session, we are unable to determine if they were actually healed by the holistic treatment. We find it extremely interesting that the size of a tumor can be reduced dramatically within a few hours of holistic treatment, when the patient is highly motivated for personal development. The reduction of tumor size is in accordance with the holistic view that many types of cancer are caused by emotional and existential disturbances. From a holistic perspective, cancer can be understood as a simple disturbance of the cells, arising from the tissue holding on to a trauma with strong emotional content. This is called “a blockage”, where the function of the cells is changed from their original function in the tissue to a function of holding emotions. The reduction of the tumor in the two cases happened when old painful emotions were identified in the tissues, in and around the tumor, and processed into understanding; when the patients finally did let go of negative beliefs and attitudes that had kept the feeling(s) repressed to that part of the body, the tumor first softened and then disappeared, presumably by apoptosis. We believe that the consciousness-based/holistic medical toolbox has a serious additional offer to cancer patients, and we will therefore strongly encourage the scientific society to explore these new possibilities. Our holistic medical research meets both ethical dilemmas and practical difficulties, as it obviously is important for the research in induced spontaneous remissions that surgery and chemotherapy is not used before it is absolutely necessary. On the other hand, is it important for the patient's survival that they receive any well-documented treatment as soon as possible. An additional aspect for the patient who is able to cure her own cancer is that she is much less likely to get cancer again and much better prepared to deal with other diseases and challenges in life. Knowing that one can fight even cancer gives a strong belief in life and the need to improve quality of life. The high incidence of secondary cancers and the physical and emotional wounds from the biomedical treatment seem to justify a focus on prevention and additional holistic treatment modules. To support the patient in learning the mastery of coherence of body and life, using the crisis of cancer to recover the human character and the purpose of life, seems turning a personal potential disaster into the greatest gift of all. When it comes down to it, life is not just about surviving; what is more important is to live fully, to learn from the great challenges of life, and to obtain the optimal quality of life while being here.

## INTRODUCTION

Cancer is one of the most common diseases of all and in modern society the second cause of death after heart disease in the adult population. Cancer can be seen as an understandable and most expected disease, because as much as the cellular order of the normal healthy multicellular organism is a sheer mystery, the break down of the order and the chaotic growth of its cells is only what one could expect from cells that lived for hundreds of millions of years as single cells on the early ocean floor. When the hidden principle that keeps all the cells in their surprising order in the body breaks down or becomes disturbed, cancer is the disease that we might most naturally expect.

The dynamic of cancer is highly complex and dependent on the original tissue from which it originates. We see a wide variety of cellular behavior. Each type of cell has specific predilections for other tissues of the body to colonialize. The pattern of growth and the rate of damage caused by the cancer will vary with the cell types. Because of this variation, many researchers find that cancer is not one disease, but hundreds of different diseases.

What we know about cancer is that it often develops over years, that dysplasia (abnormal tissue development) is often an early stage, and that carcinoma *in situ* (at site) is often found before the cancer spreads (metastasis). We also know that the order of the cell as a whole, the tissue, and the organelles of the cells are often lost at the same time, making cancer a disease characterized by a multilevel structural breakdown.

Quite surprisingly cellular order is not just lost, sometimes it is also gained and the order of the body can reappear spontaneously. This is the famous picture of spontaneous remission of cancer. How strange the concept must seem. The picture of spontaneous remission is rather well documented in the literature and the Danish researcher Ulrik Dige has shown that two out of three patients going through such a spontaneous remission experience a spiritual awakening before the remission happens[1]. When we searched on Medline (www.pubmed.gov) for "spontaneous remission cancer" we got 5,458 hits and most cancer forms are seen occasionally to disappear completely, although this is very rare. Let us define spontaneous remission of cancer as… a remission that is not caused by a biomedical treatment normally considered powerful enough to cure it. When a patient is cured with holistic medicine, we call it "induced spontaneous remission", which sounds like a paradox, but we find the meaning exact enough to use the phrase.

In general it seems that recovery of life purpose and human character is the factor most permanently connected to spontaneous remissions of cancer. It is therefore not too surprising that working on the recovery of the purpose of life and the human character[2], the essence of our holistic and consciousness-oriented medicine, can lead to spontaneous remissions of cancer.

It seems in general that the cancer cells are disappearing by apoptosis (cell death by injury or cell suicide), so a holistic cure for cancer is most likely to be successful if apoptosis can be induced. It might be a surprise for many researchers, who follow the typical biochemical way of thinking, that induction of apoptosis seems quite possible and quite natural from a holistic medical perspective as we believe that we are dealing with the same formative forces — the overall information system of the body[3,4,5,6,7,8,9,10] — in the body that induced apoptosis in embryonic life.

If we can increase the coherence of the organism[11,12,13,14,15] radically and remove the disturbances that give the cell problems with their communication, the level of information in the tissue also can be improved radically due to holistic healing[16] and the apoptosis thus induced, as apoptosis presumably happens as a function of the cells realizing that it is not a natural and sound part of the body any more. To understand this line of thinking, let us review some of our work with holistic medicine and existential healing.

## THE BASIS FOR CLINICAL HOLISTIC MEDICINE

The life mission theory[2,17,18,19,20,21,22] states that everybody has a purpose of life, which when found and realized, takes form as a huge talent. Happiness comes from living this purpose and succeeding in expressing the core talent in your life. To achieve that, it is important to develop as a person into what is known as the natural condition, a condition where the person knows himself and uses all efforts to accomplish what is most important for him.

The holistic process theory of healing[16,23,24,25] and the related quality of life theories [26,27,28] state that the return to the natural state of being is possible whenever the person gets the resources needed for existential healing. The resources needed are provided by the physician's support, usually called "holding", a well-chosen word because what the physician does here is very similar to a loving parent holding a little child. Holding has five dimensions: awareness, respect, care, acknowledgment, and acceptance; this support gives the patient the necessary resources for processing the repressed traumatic material. The processing has three dimensions: feeling, understanding, and letting go of negative attitudes and beliefs. The preconditions for holistic healing to take place are trust and the intention for the healing to take place. Existential healing is not a local healing of any tissue, but a healing of the wholeness of the person, making him much more resourceful, loving, and knowledgeable of himself, his own needs and wishes. In letting go of negative attitudes and beliefs, the person returns to a more responsible existential position and an improved quality of life. The philosophical change of the person healing is often a change towards preferring difficult problems and challenges, instead of avoiding difficulties in life[3,4,5,6,7,8,9,10]. The person who becomes happier and more resourceful often also becomes more healthy, more talented, and more able to function[26,27,28].

## SPONTANEOUS REMISSION OF CANCER — A SHORT REVIEW

Cancer is a general term for more than 100 different diseases, which all have in common that cells divide uncontrollably and form nodules that often spread and invade the surrounding tissue, such as nerves and blood vessels, which can result in a fatal outcome. In Denmark, one in three die from cancer, so cancer is one of the diseases to which we are all genetically predisposed. The question is what cancer really is and whether there are ways to prevent or treat it. Biomedical treatment is only curative in a very small group of the 100 different types of cancer, although life is often prolonged. But perhaps improved quality of life can increase the individual’s resistance to cancer, so that cancer may be prevented and treated by means of holistic medicine that effectively leads to improved quality of life? There are several indications to that effect, although there is insufficient evidence in this field.

Professor David Spiegel of the Psychosocial Treatment Laboratory at Stanford University in California is one of the great pioneers who researched the correlation between quality of life and cancer. Spiegel and his co-workers have demonstrated that women with breast cancer, who work on themselves to improve their quality of life, survive twice as long as a control group[29]. Some of the patients, who worked with themselves in his group and met once a week for a year, have survived for 10 years, while all the patients in the control group died after only 4 years (Fig. 1). The purpose of the study was to help women diagnosed with terminal cancer to improve the remaining part of their life. After 12 months, Spiegel and his co-workers demonstrated a significant improvement in the patients measured by various psychological tests: reduced pain, milder depression, less anxiety, etc. “Actually, we started looking at the 10-year survival pretty much by chance and we were very surprised by the result,” he told one of the authors (SV) on a visit at Stanford.

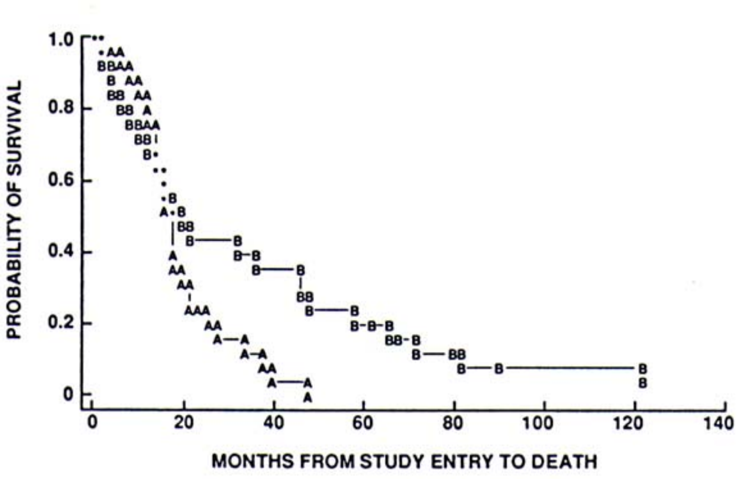

**Kaplan-Meier survival plot.**

A = control (n = 36), B = treatment (n = 50), and * = overlapping control and treatment probabilities of survival. Some points represent more than 1 case.

**FIGURE 1**. A group of women with metastatic breast cancer worked to improve their quality of life for 90 min a week for a year and on average they survived twice as long as the controls who did not work systematically to improve their lives[29].

He showed us how he worked with these patients and it was a great experience. The women shared their difficult lives with each other and their condition steadily improved as they talked about and discussed the major issues that concerned them. What can I do to improve my life? What do I need? How can I achieve that? The women in the group searched and found internal and external resources for their struggle to improve life. Spiegel has created a forum where it is acceptable to talk about the meaning of life. It was a peculiar feeling that such quiet, profound acknowledgment of the true values in life was far more valuable to health than the major emotional catharsis characteristic of many therapy courses. The way we see it, Spiegel has found a way to improve the quality of life of these women. Apparently, patients with breast cancer can do more for their own health and well being by improving their quality of life than we, as physicians, can do for them with all our medical and surgical skills. International studies, such as the one by Spiegel, indicate that improved quality of life is the best treatment available today, once the cancer has spread throughout the body. As the patients who improve their quality of life most effectively may also be assumed to be the ones to survive their cancer, there is the exciting prospect that improved quality of life would probably also be effective in preventing and perhaps even curing cancer.

The common definition of spontaneous remission is “a complete or partial, temporary or permanent disappearance of all or at least some relevant parameters of a soundly diagnosed malignant disease without any medical treatment or with treatment that is considered inadequate to produce the resulting regression[30].” A search for “spontaneous remission of cancer” on Medline (we used www.pubmed.gov) on April 20th, 2004 resulted in 5,458 references and it seems that spontaneous regression happens with almost all kinds of cancer[31], although it is reported far more often with some kinds of cancers[32,33,34,35,36,37]. A few years ago, the discussion was about the question if spontaneous remission of metastasized cancer really happened[38], as it might have been a question of misdiagnosis[39]. Today the discussion is about the possible mechanisms and how it can be induced[40] — the ideal, optimal solution to a fast killing disease. The mechanism of spontaneous remission is not clear, but apoptosis seems to be a part of it[41]. There are many speculations as to the cause of apoptosis, some believe in immunological factors[42,43], like natural killing activity[44] or antibodies [45], while others speculate on the positive effect of fever[46], thyroid hormone stimulation[47], and surgery[48,49].

The incidence is also highly controversial, from 1900 to 1960 only 176 cases were reported and some authors believe in an incidence of 1:100.000[50], while others report a number 50 times higher for some types of cancers[51]. The researcher Ulrik Dige from Denmark[1] found over 40 cases, most not reported in the literature, indicating a ratio of 1:10.000 or even higher, and a massive scientific under-reporting of the spontaneous remissions. He found that about two-thirds of the patients have undergone some kind of spiritual awakening, before the remission took place, indicating that the patient himself had an important role in the process of healing. Improving the quality of life seems to have much to do with survival and remission of breast cancer[52], but the psychological and existential elements in the process of spontaneous remission remains controversial[53,54].

The common definition seems to refer to the biomedical intervention only, as most of the spontaneous remissions (at least according to the findings of Dige[1]) seem to be induced by the patients themselves, alone or in cooperation with others. This is why we in this paper use the concept of “induction of spontaneous remission of cancer”, which of cause literally is a contradiction, but we believe that this is most correct in the light of the traditional meaning of the terms in use.

## CRITIQUE OF THE BIOCHEMICAL THEORY ON CANCER

According to the most accepted biomedical theory on cancer, the genes of the cells mutate (change spontaneously), so that over time the chromosomes in the cells become worn, damaged, and the oncogenes comes into play. An oncogene is a gene that, when mutated or expressed at abnormally high levels, contributes to converting a normal cell into a cancer cell. This leads to sick cells, which no longer fulfill their functions in the body and divide without control. We know these colonies as the cancerous tumor, which as they grow may impinge on blood vessels, nerves, or other vital structures to threaten our bodily functions and in fact our entire existence.

We believe in natural science and appreciate the contribution by medical science to the collection of a vast and complex knowledge of all the different molecular and chromosomal changes of the cells of the different cancer types. Yet the whole body of knowledge is built on the simple axiomatic understanding that the body is a piece of chemical machinery that can only be understood from the chemical level. We have given an extensive critique of this perspective and the biomedical paradigm elsewhere[3,4,5,6,7,8,9,10,55,56,57,58,59,60,61]. We believe that there exist some rare cancer forms that are caused by the genes or chromosomes, but in accordance with the lack of finding such genes related to most cancer forms, we believe that cancer is caused from our consciousness, when we repress emotions and place them in the tissues of the body[16]. We therefore also believe a cure of cancer to come from fundamental shifts in our consciousness and state of being.

If we are old and our days are numbered, cancer is no tragedy, because death is an inevitable fact of life. But often, cancer strikes — as a consequence of our poor living, in our view — long before our time should be up. What should be done naturally depends on the individual understanding and perception of cancer as a disease. If the cancerous nodule is discovered at an early stage, in many cases it can be removed completely by surgery and/or chemotherapy. This is in many cases a fairly effective solution, which most patients choose. But often the cancer reoccurs after even the most successful treatment, perhaps at an entirely different site in the body. The cancerous nodules may be a symptom of a fundamental disturbance in the body rather than an actual, local disease.

## A HOLISTIC THEORY ON CANCER

According to the holistic theory on cancer, the cells as such are not sick — although they may appear to be very sick under the microscope — but rather the entire organism is unbalanced. In order for the cells to do their job and stop dividing uncontrollably, they must receive information on what to do. According to this theory, cancer results from a disturbance in the information system, which prevents the body from providing the requisite flow of information to the cells, because of the phenomenon we refer to as a "blockage" at the particular location.

Scientific understanding of such blockages and information disturbances is still poor. In the healthy body — in our natural, living condition — all cells communicate. Cells are tiny living animals that have lived for some 3,800 million years, reproducing by division and creating perfect replicas of themselves. One thousand million years ago, these cells learned how to create the marvellously complex cell colonies that constitute man today. We consist of 200 different kinds of cells, all having the same progenitor: the fertilized egg. All cells communicate with each other and therefore know exactly what to do in their specific location.

However, sometimes things go wrong. In some areas of the body, biological order collapses and the cells start acting more and more autonomously and less and less socially — often gradually over many years. Ultimately, they act almost like free-living amoebae in the primeval sea from which we evolved. The observation that cancer does not occur at random, but rather in areas of the body where the individual has had emotional problems earlier in life — the reproductive organs, intestine, neck, etc. — is not new. However, many forms of cancer cannot be explained by this observation, as they occur throughout the body, for example in the immune system in the form of lymphatic cancer.

## BLOCKAGES MAKE THE BODY ILL

According to the holistic view, cancer occurs at sites where there is insufficient information to the cells. The work of the cells is disturbed by "blockages" in the form of painful feelings that have been repressed from the conscious to the subconscious mind through less constructive decisions in the life of the individual.

As described elsewhere[17,18,19,20,21,22], one can choose to shift one's life perspective when life hurts and in that way avoid the pain. Often we choose a life perspective that reduces our own share of the responsibility for unpleasant events. Naturally, we cannot escape responsibility in the absolute sense, but we can disclaim it subjectively. We do so by making the decisions referred to as life's "small lies", self-delusion or negative attitudes to life. What they are called is really not that important as long as we understand that they are about negative decisions contrary to life, which we make in the course of our lives to get out of difficulties and avoid the pain. Science has yet to find out why such negative decisions weaken our bodies and make us ill. The oldest explanation of this phenomenon is that, by means of our negative decisions that suppress aspects of ourselves, we split our existence into parts. The decisions are lodged in the body in such a way that our contact with this part is impaired which in turn leads to this part of the body becoming ill.

For example, if we decide that we do not care for sex, we split off our reproductive and sexual "energy", and this often gives problems in our reproductive organs and quite often in other organs in the pelvis as well when the repressed material is placed here. If we do not care for other people, we split off our ability to love (our "heart energy"), which may cause us to develop a heart condition if the repressed material is placed in the heart. The body is a complex whole, sustained by information flowing in all directions, and such split-offs are bound to have some adverse consequences for our health. In a complex manner, our negative decisions added together untie and break down the inner coherence of the body. Thus, the risk of cancer or another serious illness increases as we tell ourselves more and more lies and our biological coherence deteriorates.

Understanding the exact mechanical correlation is usually far more important to the physician than to the patient. What most patients need to understand is that improved quality of life is the road to recovery; in this way the body can restore the information flow to all its parts. Therefore, the primary goal of conversational therapy — "the quality-of-life conversation" — in cancer patients is to readjust the life perspective of the patient.

Apoptosis is extremely important to the spontaneous healing process that the holistic physician hopes to stimulate. Apoptosis is the mechanism used by medical science to explain how even patients with advanced cancer may recover miraculously, if they radically improve their quality of life. Apoptosis means that the cells self-destruct by splitting into two or three pieces, which are subsequently "devoured" by the adjacent cells. This occurs once the cells discover that they are in a location in the body where they are not supposed to be. Apoptosis is currently the object of intense research worldwide.

## HOLISTIC TREATMENT OF BREAST CANCER

The following case history from the Holistic Research Clinic in Copenhagen concerns a woman with nonmetastatic breast cancer treated according to the "feel, acknowledge, let go" concept[16]. Her case is interesting because apoptosis was apparently induced — spontaneous remission of her tumor by spontaneous cell death.

> A small, somewhat disheveled 42-year-old female enters the clinic. She sits down, panting, gives me a confused look and says: "It has just been confirmed that I have breast cancer. I am to have an operation, then radiotherapy 30 times and perhaps chemotherapy as well." One in nine women develop breast cancer, so these patients are not rare. But this particular patient was somewhat rare, because she came to me and was not willing to make do with conventional treatment. "And what do you want me to do?" I asked her, full of sympathy for her tough situation.
>
> "I would like acupuncture or something like that, I want to do something myself to fight the disease and prevent it from recurring." Indeed, the disease recurs in about 40% of patients who undergo surgery for breast cancer, although the cancer was apparently removed completely the first time. "That is a good idea," I (SV) said. "What do you expect acupuncture can do for you?" "I don't know really, perhaps I should try something else. What would you suggest?" "I suggest we work a little on your view of life and make you focus on all the good things in life, and less on your disease," I told her.

She agreed to work for an hour or so — this service is called the “quality-of-life conversation” and “healing through a shift in life-perspective” — if the process is successful and the patient succeeds in replacing her negative and bleak life perspective with a more positive one. I sensed that she was skeptical and I asked her about it. It turned out that she just finished a long-term relationship and was therefore somewhat skeptical about men in general, but after a brief talk she agreed to do some work on the couch. Here, she admitted that she really was very bitter and angry and we talked about all the harm men had done to her. Humbly I asked her to forgive all the harm inflicted on her by my gender throughout her life. That is one of my most cunning tricks, which I learned from a gestalt therapist. I shamelessly scoop up responsibility for myself, leaving only a small amount of responsibility that the patient has to take herself. The patient, whose emotional response has been arid up to now, suddenly becomes so moved by my prayer of forgiveness that tears start to flow. “I am moved,” she said. She allowed me to feel the tumor, which was clearly palpable as a 2 × 2 × 2 cm movable tumor in the lower part of her right breast. While holding the tumor, I used the same method as when locating chronic muscle tension, for example in the neck in a whiplash patient. I asked her over and over: “What feeling lies here?” She could not answer that, so I made her say: “I hate you, Daddy. I am angry with you. I give up.” I was “fishing” and tried to make her feel the negative and hostile emotions in herself, which I sensed hidden in the area around the lump.

“I have a feeling of desolation,” the patient then said. I worked for a while to make her reach beyond her emotional surface and into the deeper layer, where hopelessness and powerlessness reside, and then to continue down into the deepest layer filled with life and a belief that life will carry her. Then the patient need not take on the unbearable task of carrying her own life. We talked about surrendering to God, about the parable of the lilies of the field and the birds of the air that teaches us to believe that life is truly good and will carry us. Now and then I sensed deflation, as though the tension and alien character left the lump and it subsided and “melted” away beneath my fingers. Speechless, I once watched a video where the healer Martin Brofman turned such a tumor (the size of a hen’s egg) into something that resembled gravel and pebbles in a mere 20 minutes. Indeed, I had read Bruno Klopfer’s account from the middle of the last century where this had occurred as a case report with no scientific documentation. I had also read and heard of spontaneous remissions and of inexplicable cell death in cancerous tumors, but experiencing the tumor melting beneath my fingers was truly great. But even greater when the patient had the same experience, so I could not help but ask the patient to feel for herself whether there is a difference: “Feel the lump yourself. Is it the same, or is there a difference?” I asked her as neutrally as possible. “It feels as though it has become a little smaller,” the patient said. “How big do you think it is?” “Now it feels like it is 1.5 cm, it was bigger before.” The next time I asked her, it has become even smaller. We believe that this patient was actually ready to take responsibility and cure herself of breast cancer, but then again she had decided to have the operation. I did not contradict her and hope that they will take the trouble to carry out microscopic examination of the lump again before their final decision on radiotherapy. I can picture the pathologist writing: No confirmed malignant cells.

I talked to her on the phone a few days later. She had slept a lot, felt rather dazed since our last conversation, and she did not want to continue in my clinic. Now she just wanted to get the operation over with, as the surgeon told her that the lump was “enclosed” and could be removed in one piece, which sounded good. Nevertheless, she seemed to have grasped the essential point that life should carry her, instead of her dragging along with life. If she seizes the opportunity and learns the lesson of what happened to her, the breast cancer will probably not recur.

## SPONTANEOUS REMISSION OF TUMOR THROUGH WORK WITH LIFE PURPOSE

We (SV) have experienced another 2.5 × 2.5 cm breast tumor in a 35-year-old female, disappearing almost instantaneously when she broke through and rediscovered her life purpose[18]. The tumor, which had grown rapidly, was to be removed surgically a short time after. She participated in one of our summer courses ("Life Philosophy that Heals") and worked purposefully on herself during the course. She was a beautiful young woman who had had problems with love her entire life. When she found her purpose in life, she could see how she had denied herself all the love and kindness that she possessed. I (SV) worked with her for some sessions over a couple of hours, with the other participants forming a circle around us, watching us. During the session, I supported her breast with the lump to help her confront the difficult feelings buried in the tissue.

It was a very loving session and a moving scenario as she ascertained with great surprise that the tumor grew smaller and softer in the course of the session and we were all close to tears, when at the end of the last session she found almost in a state of shock that not only had the tumor gone soft, it had become as small as a "sunflower seed" and was very difficult to locate. The tumor worked and changed beneath my hands during the session. It became less hard as she took responsibility for her pain and explored her fundamental, past feeling of being helpless and unloved. During the sessions, she searched for her fundamental denials of her life purpose and she found what she was searching for. A number of negative decisions that she had made during her life and could now let go of. She healed and the tumor vanished almost completely. The remainder of the tumor was surgically removed the following week.

This story about remission of the tumor following acknowledgment of the life purpose illustrates the purpose of life theory of holistic medicine. Another participant at the course was asked to describe her experience.

> **JM, participant in "Life Philosophy that Heals"[28], Course 2, 2002, wrote:** One of the participants at the course was a woman, NN, with an extremely good-looking and loving appearance. From the time when I first saw her, I thought she had a delicate, angelic appearance, and she was definitely a person that I considered to be very "sweet". Actually, I sensed that on the one hand NN was extremely delightful, loving and warm, but on the other dismissive, calculating and cool.
>
> About halfway into the course, NN told us that when she returned from the course she was going home to have a lump in her right breast removed. She told us that the tumour was very hard and about the size of a hazelnut. She continued to say that she is very nervous about the operation and scared. She said that she joined the course, because she did not believe in what the conventional health care system has to offer. Also, she wanted to find out, whether SV could help her.
>
> Then SV asked NN, if she wanted him to work with her. She did and SV asked all of us whether we felt it was allright for him/us to spend some of the afternoon working with NN. SV suggested that he/we spend about 3 or 4 periods of 45 minutes on NN's lump. Everybody agreed that it was the most important thing right now and naturally he/we should work with NN, since she felt like doing it now. Then we placed some quilts in the middle of the floor and NN lied down on them, while SV held her head in his lap. The rest of us sat down on the floor around them, but not too close. SV asked NN if he could feel her breast and if she would show him where the lump was. NN agreed to that and she located the tumour, which according to SV was 2.5 × 2.5 × 2.5 cm. SV spoke very softly and tenderly to NN and an atmosphere of tenderness, care and love spread around them. Before long NN looked like a little girl cared for by her father. SV felt and held the lump. Then he started massaging it, while asking NN what feelings were located in her breast and what feelings were in the lump. NN starts to cry, as I remember it, like a little girl.
>
> NN said that she was always required to be 'big'. She talked about the time when her mother died, NN was about 13 or 14 years old and how her father was very incoherent

> afterwards and how NN became the one who kept herself and her father together. She said that she had not mourned or cried about the loss of her mother, since there simply was no room for that and how she had run the house for her father and thereby assumed her mother's role. She became her father's wife. NN cried in the process, and SV massaged her, while meta-communicating with her, assuring her that it was fine for him/us to spend time on working with her lump. That nobody felt that she was taking time away from us, neither causing any inconvenience or trouble. The reason that SV made a point of emphasising this was that NN seemed to find it difficult to relax. It seemed as though NN felt that it was not worth spending any time on her and her lump. As though she cannot believe and accept that, we all felt that she should have that kind of attention and love.
>
> SV asked her about that, and she explained how she always found it difficult to accept attention and care. NN said that, in fact, she was never able to accept it, except when she was ill. The result was that now and then she would be ill, during which time she would then receive and be able to accept love. That was NN's pattern: illness means love.
>
> While massaging and working with NN, SV asked her to feel the lump now and then. The more NN acknowledged her situation, the softer the lump seemed to become and when NN realised that ill means love, the lump also began to diminish.
>
> In the evening, following 40 to 45 minutes of work on NN's tumour, he asked her to locate it and give an estimate of its size. To begin with NN actually had difficulty locating it, she was clearly very moved, she was crying tears of joy, whereupon she told us that now she thought it was the size of a sunflower seed. She had signs of relief, both in her voice and in her eyes. She had managed to give herself up to the process, whereupon her tumour had become softer and then smaller. She had received attention, respect, love and could now go home with a tumour the size of a sunflower seed. All in all, it was an extremely beautiful, quiet and very moving experience and I am very grateful for having had the opportunity to witness it.

From the point of view of holistic medicine, what happens is that, as discussed in the previous chapter, the malignant cells self-destruct (apoptosis), when the blockage is removed. The information relationships in the tissues are restored and the cells suddenly discover that they are growing in a place in the body, where they are not supposed to be.

## DISCUSSION

Every physician must treat his patient according to his knowledge and his consciousness with the intention to help the patients as much as possible, and if possible, cure them. The first and most important medical law ever since Hippocrates (460–377 BCE) is "First, do no harm". Harming a patient includes not keeping up one's knowledge of best practice; for instance, not knowing diagnostic practices and the documented cures of a disease. But even when you know how to diagnose and which cures and treatments are available, things are often complicated when it comes to cancer. Often, the cures are not too good and the drugs have very high Number Needed to Treat (NNT) of often 5, 10, and even 20 or more and the surgical procedures are often not efficient in preventing the disease from coming back, which often happens with terrifying incidences (up to 30% of patients with a positive family history for breast cancer operated themselves for a breast cancer will have tumor recurrence[62] and when there are metastasis to lymph nodes, the 10-year survival rate is only 56%[63]). This means that the offer to many cancer patients is not really good.

We know that spontaneous remission of cancer is seen with almost all kinds of cancer and we know that it often happens after a spiritual breakthrough. The spiritual breakthrough is almost always about being more alive, knowing oneself and the purpose of life better, stepping fully into personal character, realizing talents and how to use them. All of this is what holistic medicine aims at supporting the patient to achieve. This means that we have a chance of inducing this “spontaneous” remission, making the patient not only well, but also much more happy, wise, and with a life. Improving the quality of life is the “name of the game” and we believe quality of life to be the best medicine there is. The chance of inducing the remission must be only 5% (as it was in the famous study by Spiegel[29]), but it might be that our new understanding of recovery of character and purpose of life makes the fraction much higher.

How can we find out how powerful our holistic medical tools are when it comes to cancer? Only by trying to heal the patients and whenever we see a radical reduction of a tumor by believing it and daring to wait and see what happens: Will it come back or will it disappear completely? The experiment is highly risky, as we risk that a patient who could have survived with immediate surgery gets metastasis and dies as a consequence of the experiment. But then again, if the patient gets the surgery, what is the chance for a recurrence after this treatment? And what if the chance for a recurrence of the tumor disappeared completely because this patient learned from the disease and reorganized her life, including all values, philosophy of life, lifestyle, etc? What the patient has gained in the later case is often described as something of tremendous value, subjectively valuable even enough to justify getting the disease in the first place.

If the patient is an adult, s/he must take every opportunity into consideration and make up his or her own mind about what is the optimal and appropriate treatment or combination of treatments. In the end, the decision must be made by the patient. But as we know how difficult a situation it is to be seriously sick and how often the patient just follows the recommendation of the physician out of fear, we as physicians cannot escape our responsibility that easy. We need to be clear about our own position in order to be able to guide the patients.

Sometimes the patient has several physicians and if they work within different medical paradigms[56] they are likely to give the patients different advice and even to judge and condemn the treatment given by the physician from the different paradigms. The problem becomes even more complicated if there is a documented biomedical treatment, say with a 10-year survival rate of 50%, and another treatment, say consciousness-based, which is undocumented as most of these treatments unfortunately are due to the sad contemporary lack of research funding. So the research is practically only carried out with single patients, like the two cases reported in this paper. So how can this research best be done? And what are the ethical dilemmas involved? Obviously, the patient will benefit tremendously from her different physicians giving her the freedom to choose her own treatment and not to push her, but just inform her honestly and objectively. The research would benefit tremendously from that as well.

In many cases, the combination of the biomedical and the holistic treatment is very simple and without any problems at all. If the cancer has metastasized to the lung or liver, there is no effective cure and the oncologist will fully accept that the patient tries any kind of alternative treatment. If the patient has already decided not to get the biomedical treatment of chemotherapy or surgery and come to the holistic physician, the situation is in principle simple, but in this case the holistic physician is at risk of being accused of giving an alternative that cheats the patient to feel that there is a real choice, when only the biomedical treatment is documented. In this case, it would be fair to look at the circumstances before judging: Is the alternative therapist a physician knowing the disease of the patient and the biomedical treatments and their effect? Is he doing research, systematizing his efforts, explaining in a scientific way what he is doing? If the patient is on a waiting list for treatment, or if the tumor is small and the risk of metastasis is known to be limited, this is also an important aspect to consider in favor of allowing the patient to try alternative treatments, while there is time.

From our perspective, there is so much theory and so many useful techniques in holistic medicine/consciousness-based medicine that this should be taken seriously by biomedical physicians also so that what might be a very important scientific development is not hindered because of sheer conservatism in the medical community. As our consciousness-based medicine has not been clinically tested yet, we fully understand skepticism. But as we believe that recovery of the human character and the purpose of life has a chance of being developed into a cure or supplementary cure for the segment of patients motivated to work with themselves, we believe it should be tested. Also because such a cure for cancer might be suspected to be highly effective in preventing cancer in the long run and at the same time dramatically improve the quality of life, general health, and ability of the patient, we feel obliged to test it in the clinic. We are obliged to get full consent of the patients, after having informed them that they participate in an experiment and that there is a high risk of having no effect at all on the disease.

We believe that we are ethically obliged to take our great medical science further and develop new treatments where the old ones have failed or are insufficient as is often the case with biomedical treatments of cancer. We believe in the new medicine as we have already seen so much good happen to our patients using it with a variety of diseases. We are at the beginning of using it and we do not know very much about its effects or side effects and we will undoubtedly make some mistakes while experimenting. In spite of our very best efforts not to do harm, some patients might even die because of choosing our offer instead of the traditional biomedical treatment, which we would regret bitterly. But we believe that we, in the end, will provide the world with a complementary, efficient tool for curing at least a part of the most motivated fragment of the cancer patients. As we see it, this cure will have very few and very limited negative side effects with a lot of positive impact on the quality of life, general health, and ability of the patients. This is our hope and belief, but we beg forgiveness, if we in the future come to make the patients suffer because of it.

## CONCLUSION

The recovery of human character and purpose of life with consciousness-based medicine seems efficient in inducing spontaneous remissions of many different diseases[2, 19, 20, 21, 22, 54, 55, 56, 57, 59, 60, 65, 66, 67, 68, 69, 70]. On two different occasions, we observed breast tumors reduced to less than half their original diameters (clinically judged) during a holistic session, while working with the patients in accordance with the holistic process theory of healing, the life mission theory, and the theory of human character. One of the tumors was histologically diagnosed as malignant breast cancer prior to the session, the other was under examination. As both patients had the affected regions of the breast surgically removed immediately after the session, we do not know if they were actually healed by the holistic treatment.

We find it extremely interesting that the size of the tumor can be reduced dramatically within a few hours of holistic treatment, when the patient was highly motivated for personal development. The reduction of tumor size is in accordance with the holistic view that many types of cancer as caused by emotional and existential disturbances. From a holistic perspective, cancer can be understood as a simple disturbance of the cells, arising from the tissue holding on to a trauma with strong emotional content, in what we call “a blockage”, allocating the function of the cells from their original function in the tissue to a function of holding emotions. The reduction of the tumor happened when old painful emotions were identified in the tissues, in and around the tumor, and processed into understanding; when the patients finally did let go of their negative beliefs and attitudes that kept the feeling(s) repressed to that part of the body, where the tumor first softened and then disappeared, presumably by apoptosis.

We believe that the consciousness-based/holistic medical toolbox has a serious offer to cancer patients, and we will therefore strongly encourage the scientific society to explore these new possibilities. Our holistic medical research meets both ethical dilemmas and practical difficulties as it obviously is important for the research in induced spontaneous remissions that surgery and chemotherapy is not used before it is absolutely necessary; on the other hand, is it important for the patient’s survival that they receive any well-documented treatment as soon as possible. An additional aspect is that if the patient can cure her own cancer, she is much less likely to get cancer again and much better prepared to deal with other diseases and challenges in life. Knowing that one can fight even cancer gives a strong belief in life and in our own power to improve the quality of life. The high incidence of secondary cancers and the physical and emotional wounds on many cancer patients under biomedical treatment seem to justify a focus on prevention.

To support the patient in learning the mastery of coherence of body and life, using the crisis of cancer to recover the human character and the purpose of life, seems turning a personal potential disaster into the greatest gift of all. When it comes down to it, life is not just about surviving. What is more important is to live life fully, to learn from the great challenges of life, and to obtain the optimal quality of life while being here.

## ACKNOWLEDGMENTS

This study was supported by grants from IMK Almene Fond. The quality of life research was approved by the Copenhagen Scientific Ethical Committee under number (KF)V.100.2123/91.

## BIOSKETCHES

**Søren Ventegodt, MD,** is the Director of the Quality of Life Research Center in Copenhagen, Denmark. He is also responsible for a Research Clinic for Holistic Medicine in Copenhagen and is a popular speaker throughout Scandinavia. He has published numerous scientific or popular articles and a number of books on holistic medicine, quality of life, and quality of working life. His most important scientific contributions are the comprehensive SEQOL questionnaire, the very short QoL5 questionnaire, the integrated QOL theory, the holistic process theory, the life mission theory, and the Danish Quality of Life Research Survey, 1991–94 in cooperation with the University Hospital of Copenhagen and the late pediatric professor Bengt Zachau-Christiansen. E-mail: ventegodt@livskvalitet.org. Website: http://www.livskvalitet.org

**Mohammed Morad, MD**, is Specialist in Family Medicine, Lecturer in Family Medicine at the National Institute of Child Health and Human Development, Division of Community Health, Ben Gurion University of the Negev and the Medical Director of a large area clinic in the city of Beer-Sheva. He has publications on Bedouin health, health aspects, spiritual health, and aging in persons with intellectual disability, and is a presenter on topics such as health policy and services for the disadvantaged at national and international conferences. E-mail: morad62@barak-online.net

**Eytan Hyam, MD**, is a specialist in family medicine and the Director General of the Soroka University Medical Center, Clalit Health Services, Faculty of Health Sciences, Ben Gurion University, Beer-Sheva, Israel. E-mail: eytanh@clalit.org.il

**Joav Merrick, MD, DMSc,** is Professor of Child Health and Human Development affiliated with the Zusman Child Development Center, Division of Pediatrics and Community Health at the Ben Gurion University, Beer-Sheva, Israel; the Medical Director of the Division for Mental Retardation, Ministry of Social Affairs, Jerusalem; and the Founder and Director of the National Institute of Child Health and Human Development. He has numerous publications in the field of child and human development, rehabilitation, intellectual disability, disability, health, welfare, abuse, advocacy, quality of life, and prevention. Dr. Merrick received the Peter Sabroe Child Award for outstanding work on behalf of Danish Children in 1985 and the International LEGO-Prize ("The Children's Nobel Prize") for an extraordinary contribution towards improvement in child welfare and well being in 1987. E-mail: jmerrick@internet-zahav.net. Website: www.nichd-israel.com
